# Assessment of safety margin after microwave ablation of stage I NSCLC with three-dimensional reconstruction technique using CT imaging

**DOI:** 10.1186/s12880-021-00626-z

**Published:** 2021-06-07

**Authors:** Peng Yan, An-na Tong, Xiu-li Nie, Min-ge Ma

**Affiliations:** 1 Department of Oncology, Jinan Central Hospital Affiliated to Shandong University, Jinan, People’s Republic of China; 2 Department of Radiation, The 960th Hospital of the PLA Joint Logistics Support Force, Jinan, People’s Republic of China; 3 Department of Radiology, Jinan Central Hospital Affiliated to Shandong University, Jinan, People’s Republic of China; 4 Department of Radiology, The Affiliated Hospital of Qingdao University, Qingdao, People’s Republic of China

**Keywords:** Lung cancer, Microwave ablation, Ablative margin, Three-dimensional reconstruction

## Abstract

**Objective:**

To assess the ablative margin of microwave ablation (MWA) for stage I non-small cell lung cancer (NSCLC) using a three-dimensional (3D) reconstruction technique.

**Materials and methods:**

We retrospectively analyzed 36 patients with stage I NSCLC lesions undergoing MWA and analyzed the relationship between minimal ablative margin and the local tumor progression (LTP) interval, the distant metastasis interval and disease-free survival (DFS). The minimal ablative margin was measured using the fusion of 3D computed tomography reconstruction technique.

**Results:**

Univariate and multivariate analyses indicated that tumor size (hazard ratio [HR] = 1.91, *P* < 0.01; HR = 2.41, *P* = 0.01) and minimal ablative margin (HR = 0.13, *P* < 0.01; HR = 0.11, *P* < 0.01) were independent prognostic factors for the LTP interval. Tumor size (HR = 1.96, *P* < 0.01; HR = 2.35, *P* < 0.01) and minimal ablative margin (HR = 0.17, *P* < 0.01; HR = 0.13, *P* < 0.01) were independent prognostic factors for DFS by univariate and multivariate analyses. In the group with a minimal ablative margin < 5 mm, the 1-year and 2-year local progression-free rates were 35.7% and 15.9%, respectively. The 1-year and 2-year distant metastasis-free rates were 75.6% and 75.6%, respectively; the 1-year and 2-year disease-free survival rates were 16.7% and 11.1%, respectively. In the group with a minimal ablative margin ≥ 5 mm, the 1-year and 2-year local progression-free rates were 88.9% and 69.4%, respectively. The 1-year and 2-year distant metastasis-free rates were 94.4% and 86.6%, respectively; the 1-year and 2-year disease-free survival rates were 88.9% and 63.7%, respectively. The feasibility of 3D quantitative analysis of the ablative margins after MWA for NSCLC has been validated.

**Conclusions:**

The minimal ablative margin is an independent factor of NSCLC relapse after MWA, and the fusion of 3D reconstruction technique can feasibly assess the minimal ablative margin.

**Supplementary Information:**

The online version contains supplementary material available at 10.1186/s12880-021-00626-z.

## Introduction

Worldwide, lung cancer has the highest incidence and mortality of all cancers, with 2.1 million new lung cancer cases and 1.8 million deaths in 2018 [1]. Lung cancer has been divided into two main histological types: small cell lung cancer (SCLC) accounts for 15–25% of all lung cancer cases, and non-small cell lung cancer (NSCLC) accounts for approximately the remaining 75–85% [2]. As low-dose CT screening has become more widespread, more early-stage lung cancers have been screened [3].

Surgical resection remains the cornerstone of therapy for stage I NSCLC. Lobectomy with hilar and mediastinal lymphadenectomy is the standard surgical treatment for stage I NSCLC. The five-year survival rate has been reported to be between 57 and 85% [4, 5]. However, approximately 20% of patients with early-stage NSCLC are unable to tolerate surgery because of compromised cardiopulmonary functions or other comorbidities [4].

Stereotactic body radiation therapy (SBRT) is an option for inoperable stage I NSCLC patients [6–8]; however, SBRT has a risk of leading to radioactive lung damage and decreased cardiopulmonary functions [9, 10].

In recent years, a number of studies have shown that thermal ablation is a safe, feasible, and effective treatment for medically inoperable stage I NSCLC patients [11–15]. Microwave ablation (MWA) is a thermal ablation therapy that has been widely applied during the last decade in various solid tumors. MWA has the advantage of having a larger ablation zone and less heat sink effect than radiofrequency ablation (RFA) [16]. Based on these advantages, MWA is the preferred technique in the ablation of NSCLC.

Some studies have shown that an adequate ablative margin is an independent risk factor for complete ablation [17–19]. Because malignant tumors are characterized with irregular pattern of growth, how to determine completely ablation is crucial. Studies demonstrated the ablative margin greater than 5 mm had achieved complete ablation [18, 25]. But in practice, it was difficult to precisely assess the safety margin by conventional 2D CT scan. The purpose of this study was to investigate the results of three-dimensional (3D) reconstruction software for evaluating the minimal ablative margin. We evaluate whether fusion of 3D reconstruction has an advantage over CT axial contrast.

## Methods

### Patients

We reviewed all stage I NSCLC patients who underwent treatments at our institution between January 2015 and October 2018. We drafted the inclusion and exclusion criteria.

Eligible patients were enrolled according to the following criteria: (1) histological diagnosis of squamous cell carcinoma or adenocarcinoma; (2) complete clinical and histological information as well as follow-up data; (3) age > 18 years old; (4) no previous treatment for cancer; and (5) clinical stage I disease (TNM staging system, eighth edition of the American Joint Committee on Cancer).

The exclusion criteria were as follows: (1) tumors with a location close to the hilus pulmonis; (2) patients had other malignancies; (3) systemic infection, autoimmune disease or inflammation; (4) Eastern Cooperative Oncology Group (ECOG) score ≥ 3; and (5) platelets < 5 × 10^9^/L or coagulation dysfunction indicated by an international normalized ratio (INR) > 1.5.

Finally, 36 patients remained and were analyzed in the study.

#### Microwave ablation

The patients fasted for 6 h before the MWA procedure. Half an hour before the procedure, patients were given breathing training and 30 mg codeine tablets orally to avoid coughing during the procedure.

Local anesthetic was applied for each patient at the puncture site using 1% lidocaine. During the procedure, monitoring of the patient heart rate, continuous electrocardiogram, oxygen saturation, and blood pressure were performed. All complications resulting from the ablation procedure were recorded and classified in accordance with the guidance of the Society of Interventional Radiology [20].

The patients were treated with MWA under computed tomography guidance (Brilliance 16, Philips Electric). An ECO-100C MWA (ECO Microwave Electronic Institute, Nanjing, China) system was used. The microwave emission frequency was 2,450 ± 50 MHz, and the output level adjustable continuous wave ranged from 0 to 150 W. For the microwave antenna, the effective length was 100–180 mm, and the outside diameter was 14–20 G, with a 15 mm active tip. The surface temperature of the antenna was cooled with a water circulation cooling system. The ablation power was selected as 40–50 W with a 5–10 min duration. The ablation zone was nearly 2.5 × 3 cm for MWA, and the output was 40–50 W/5–10 min. Two ablation antennae were applied together for tumors larger than 3.0 cm. A CT scan was performed immediately after the MWA procedure to assess the ablative margin as well as to observe immediate complications. Technical success was defined as complete coverage of the tumors by the ablation zone on CT [21]. The exudate area around the tumor is at least 5 mm out of the tumors margin. If not, the ablation duration would be prolonged.

The procedures were performed by doctors with more than 5 years of experience in tumor ablation.

#### Three-dimensional reconstruction and fusion

We performed three-dimensional reconstruction using the original images from 1 month after ablation. 3D reconstruction software (Lung Nodule assessment, Philips, Netherlands) was applied to reconstruct the 3D images and to measure the ablative margin in various directions. All the procedures were performed by a radiographer with over 5 years of experience in 3D image reconstruction who was blinded to the purpose of the trial.

The CT data, which had 1.0-mm intervals as DICOM files, were imported into the workstation (Extended Brilliance Workspace V4.5.5.51035, Philips, Netherlands) following the instructions for the procedure: The nodules contours were automatically delineated by the system, and then manually corrected in axial, sagittal, and coronal positions. Once correction was complete, the reconstruction program was initiated. Background was removed by adjusting window width and window level. Pre- and postablation 3D reconstruction images were fused, and then rotated fused images in various directions to observe the minimal ablative margin.

The minimal ablative margins were recorded by the radiologist. Each case was reconstructed and analyzed within 15 min.

#### Follow-up

The patients were followed with contrast-enhanced CT within 7 days, in the first month after the procedure, every 3 months after the procedure until either local tumor progression (LTP) or new lesions appeared, and every 6 months beginning in the 3rd year. For patients with cardiac insufficiency or elderly patients, we chose plain CT for follow-up. The local tumor progression interval was defined as the interval from the date of treatment initiation to the date of LTP. Local tumor progression was defined as contiguous enlargement or a change in the shape of the ablation zone or the development of contrast enhancement in part of the zone [22–24]. The distant metastasis (DM) interval was defined as the interval from the date of treatment initiation to the date of new lesions appearing in the lung or other organs.

Disease-free survival (DFS) was defined as the interval from the date of treatment initiation to the date of LTP, distant metastases or death. Follow-up data collection was terminated in October 2019.

#### Statistical analysis

A Cox proportional hazards model was applied to explore the risk factors for LTP interval and DFS, with proportional hazard ratio (HR) and the calculated 95% confidence intervals (CIs). Receiver operating characteristic (ROC) curves analysis to assess the minimal ablative margin on local recurrence. The LTP interval, DM interval, and DFS were calculated and depicted using the Kaplan–Meier method and compared using the log-rank test. The correlation between the ablative margins and other baseline characteristics tested by Pearson correlation analysis. *P* < 0.05 was regarded as statistically significant. All statistical analyses were conducted with SPSS Statistics 25 (IBM Corporation, NY, USA).

## Results

### Baseline characteristics

From January 2015 to October 2018, a total of 36 patients with stage I NSCLC were enrolled based on the inclusion and exclusion criteria. The baseline characteristics are summarized in Table 1. Among these 36 patients, 21 (58.3%) patients were male, and 15 (41.7%) were female, with ages from 60 to 91 (mean 74.4 ± 7.0). Adenocarcinoma was pathologically diagnosed in 27 patients (75.0%), and squamous cell carcinoma was pathologically diagnosed in 9 patients (25.0%). Based on the TNM staging system, 32 patients presented with stage IA disease (T1N0M0), followed by 4 with stage IB disease (T2aN0M0). By the end of the follow-up, three patients were confirmed to be dead.

**Table 1:** Baseline characteristics of the enrolled patients

Characteristics	Number of patients
Overall	36
*Age (years)*	
< 70	13
≥ 70	23
Mean ± SD	74.44 ± 8.46
Range	60–91
*Gender*	
Male	21
Female	15
*ECOG score*	
0	10
1	20
2	6
*Histological types*	
Adenocarcinoma	27
Squamous cell carcinoma	9
*T stage*	
T1a	12
T1b	15
T1c	5
T2a	4
*Tumor size*	
0.5–1 cm	13
1.1–2 cm	15
2.1–3 cm	5
3.1–4 cm	3
*Clinical stage*	
IA	32
IB	4
*Ablative margin*	
< 5 mm	18
≥ 5 mm	18
Mean ± SD	0.51 ± 0.17
Range	2.8–8.8 mm

*ECOG* Eastern Clinical Oncology Group

#### Risk factor analysis for patients

**Fig. 1:**
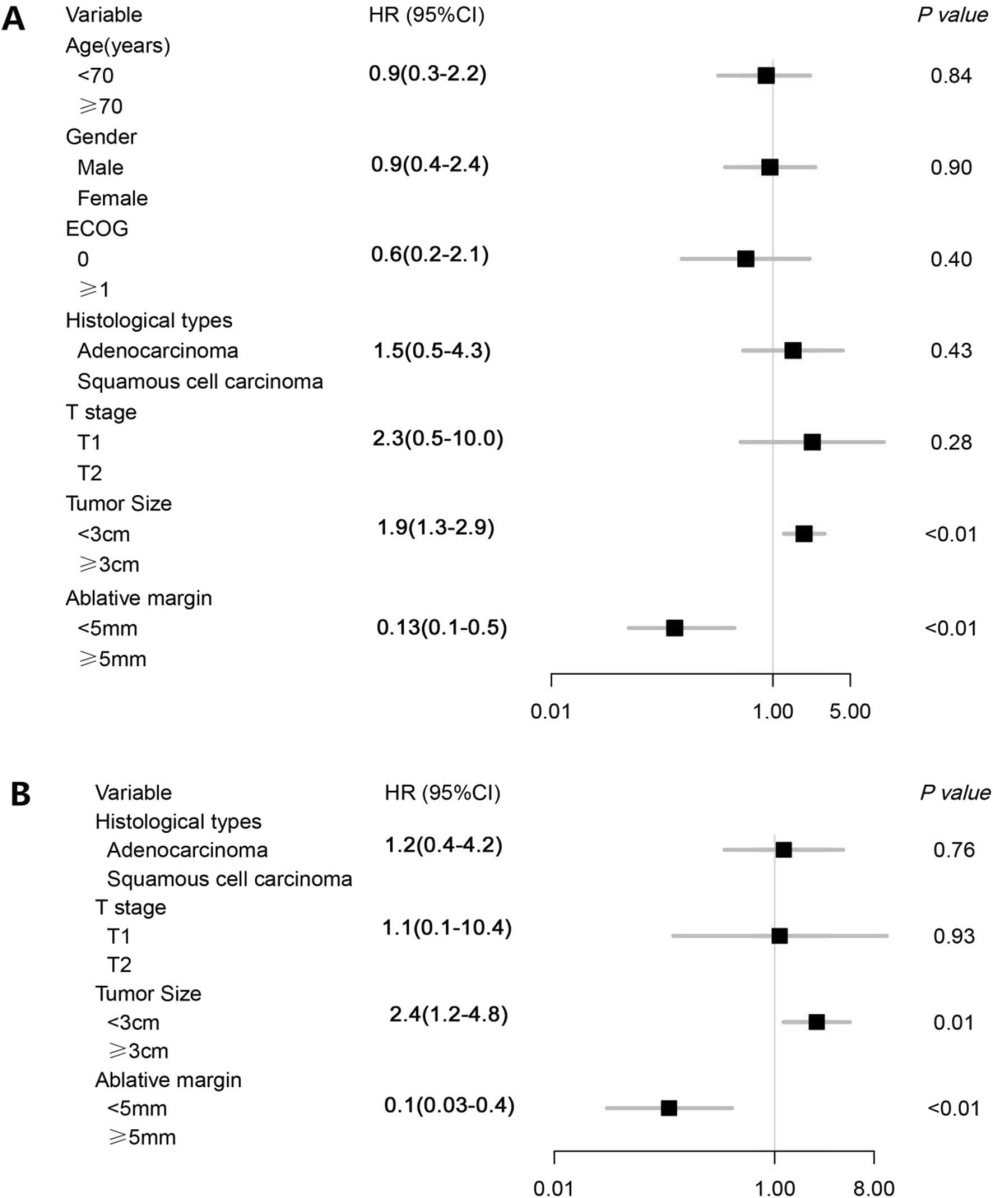
Results of univariate analysis (**a**) and multivariate analysis (**b**) for the association of clinical characteristics with LTP interval

**Fig. 2:**
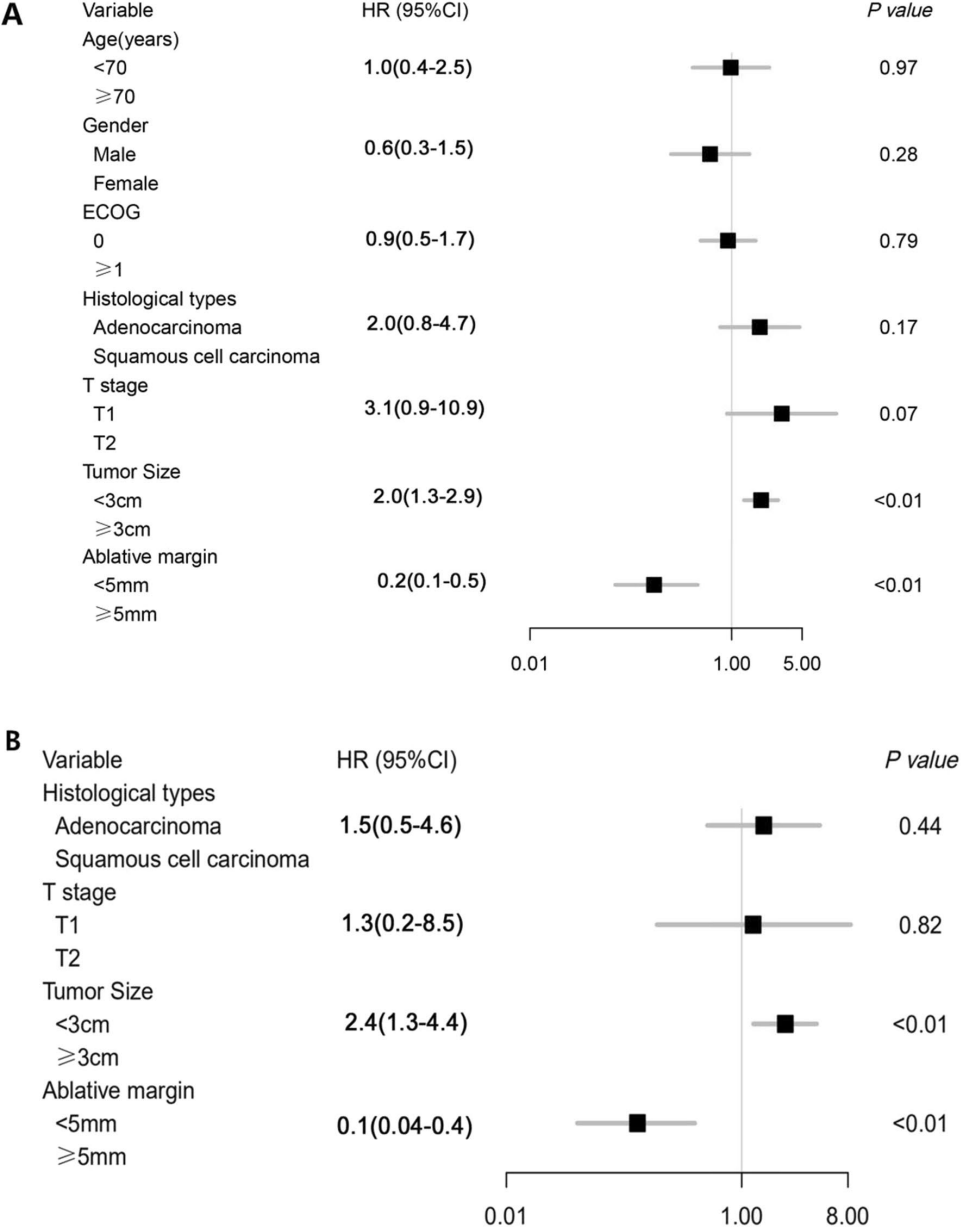
Results of univariate analysis (**a**) and multivariate analysis (**b**) for the association of clinical characteristics with DFS

The relationships among the LTP interval, DFS and clinical characteristics are shown in Figs. 1 and 2.

In the univariate Cox regression analysis, tumor size (HR = 1.91, *P* < 0.01) and minimal ablative margin (HR = 0.13, *P* < 0.01) were independent prognostic factors for LTP interval.

The variables that were statistically significant in the univariate analysis and those that we thought were related to the LTP interval were included in the multivariate Cox regression analysis. Multivariate analysis indicated that tumor size (HR = 2.41, *P* = 0.01) and minimal ablative margin (HR = 0.11, *P* < 0.01) were independent prognostic factors for the LTP interval.

In the univariate Cox regression analysis, tumor size (HR = 1.96, *P* < 0.01) and minimal ablative margin (HR = 0.17, *P* < 0.01) were independent prognostic factors for DFS.

Likewise, the variables that were statistically significant in the univariate analysis and those that we thought were related to DFS were included in the multivariate Cox regression analysis. Multivariate analysis indicated that tumor size (HR = 2.35, *P* < 0.01) and minimal ablative margin (HR = 0.13, *P* < 0.01) were independent prognostic factors for DFS.

#### The optimal cut-off value for ablative margin

**Fig. 3:**
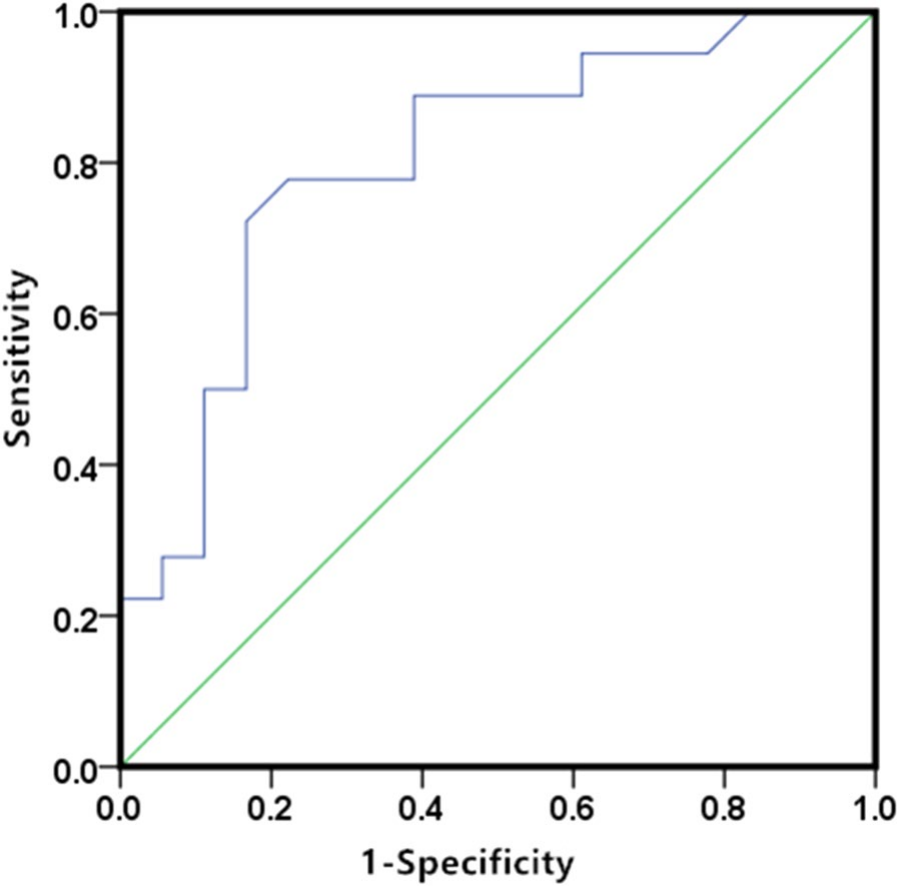
ROC curves analysed the minimal ablative margin on local recurrence

ROC curve was generated to assess the ability of the ablative margin to predict the local recurrence. The optimal cut-off value was 4.9 mm (Fig. 3). We divided the patients into two groups based on an approximate ablative margin of 5 mm.

#### LTP interval, DM interval and DFS in different groups

**Fig. 4:**
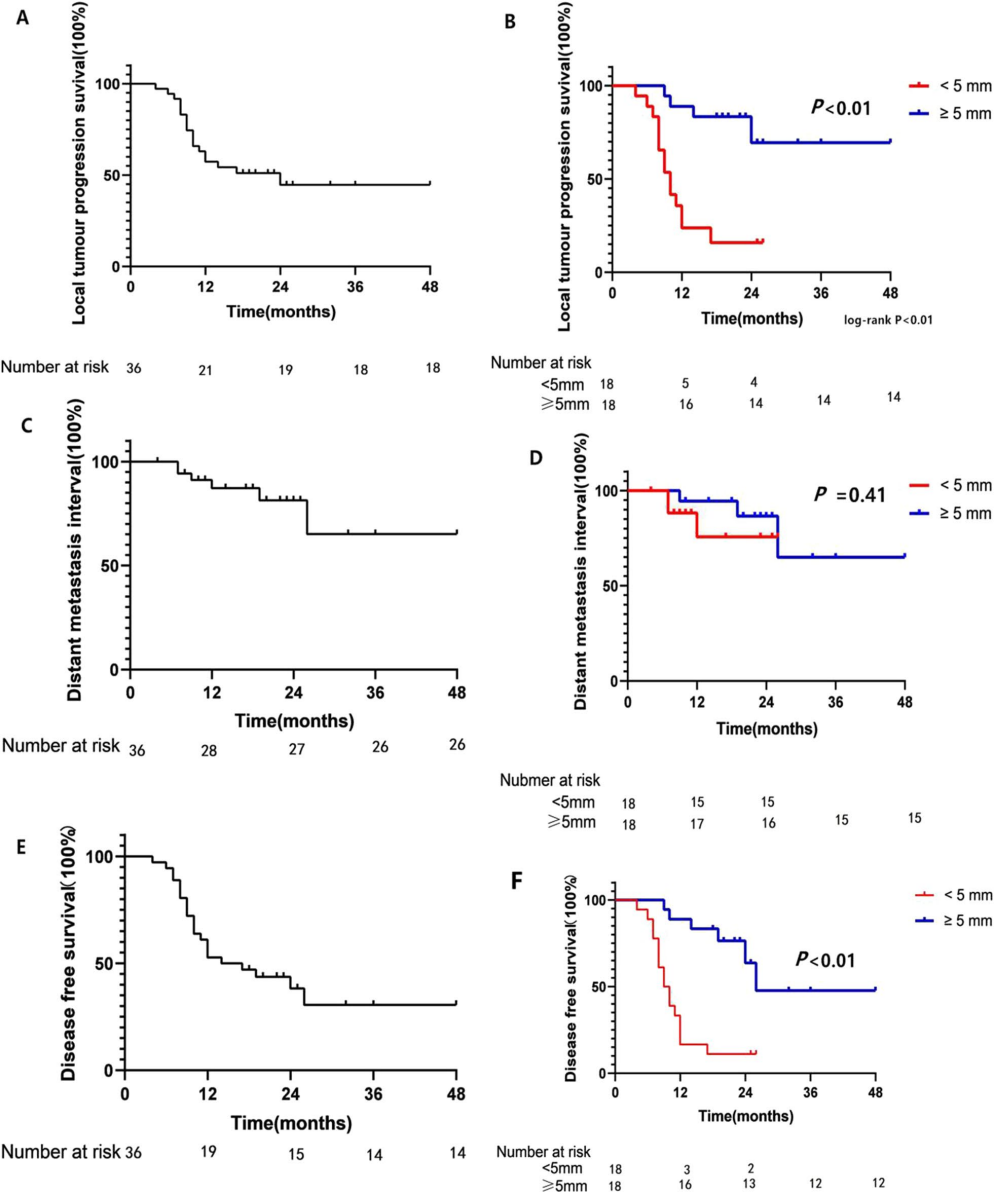
Kaplan–Meier survival curves of LTP interval in all patients (**a**) and in patients with a minimal ablative margin < 5 mm and ≥ 5 mm (**b**). Kaplan–Meier survival curves of DM interval in all patients (**c**) and in patients with a minimal ablative margin < 5 mm and ≥ 5 mm (**d**). Kaplan–Meier survival curves of DFS in all patients (**e**) and in patients with a minimal ablative margin < 5 mm and ≥ 5 mm (**f**)

The LTP interval, DM interval and DFS in different groups are shown in Fig. 4.

The LTP interval and DFS of the patients in the < 5 mm group were significantly shorter than those in the ≥ 5 mm group (*P* < 0.01). The DM interval of the patients was not significantly different between groups.

As shown in Table 2, in all patients, the 1-year and 2-year local progression-free rates were 57.3% and 44.7%, respectively. In the group with a minimal ablative margin < 5 mm, the 1-year and 2-year local progression-free rates were 35.7% and 15.9%, respectively. In the group with a minimal ablative margin ≥ 5 mm, the 1-year and 2-year local progression-free rates were 88.9% and 69.4%, respectively.

**Table 2:** LTP interval, DM interval and DFS for patients

Survival outcomes	Survival analysis								
	LTP-free rate (%)			DM-free rate (%)			DFS rate (%)		
	Total	< 5 mm	≥ 5 mm	Total	< 5 mm	≥ 5 mm	Total	< 5 mm	≥ 5 mm
1-year	57.3	35.7	88.9	87.2	75.6	94.4	52.8	16.7	88.9
2-year	44.7	15.9	69.4	81.4	75.6	86.6	38.2	11.1	63.7

In all patients, the 1-year and 2-year distant metastasis-free rates were 87.2% and 81.4%, respectively. In the group with a minimal ablative margin < 5 mm, the 1-year and 2-year distant metastasis-free rates were 75.6% and 75.6%, respectively. In the group with a minimal ablative margin ≥ 5 mm, the 1-year and 2-year distant metastasis-free rates were 94.4% and 86.6%, respectively.

In all patients, the 1-year and 2-year disease-free survival rates were 52.8% and 38.2%, respectively. In the group with a minimal ablative margin < 5 mm, the 1-year and 2-year disease-free survival rates were 16.7% and 11.1%, respectively. In the group with an ablative margin ≥ 5 mm, the 1-year and 2-year disease-free survival rates were 88.9% and 63.7%, respectively.

#### The correlations between patient characteristics and the ablative margin

The relations between the minimal ablative margin and other characteristics (age, gender, ECOG, histological types, T stage, tumor size) are shown in Table 3. Negative correlations were found between ablative margin and tumor size (*r* = − 0.37, *P* = 0.03).

**Table 3:** The correlations between patient characteristics and the minimal ablative margin

	Minimal ablative margin	*P* value
Age	0.06	0.74
Gender	0.17	0.32
ECOG	− 0.08	0.65
Histological types	− 0.19	0.26
T stage	− 0.31	0.06
Tumor size	− 0.37	0.03*

*P < 0.05

#### Complications

There were no treatment-related deaths. Common complications in the two groups of patients were fever, pain, pneumothorax, and pleural effusion (shown in Table 4).

**Table 4:** Complication for patients

Ablative margin < 5 mm (n = 18)		Ablative margin ≥ 5 mm (n = 18)	
Major	Minor	Major	Minor
Infection (n = 1)	Pain (n = 4) Fever (n = 3) Pneumothorax (n = 7) Pleural effusion (n = 3)	Bronchopleural fistula (n = 1)	Pain (n = 3) Pneumothorax (n = 4) Pleural effusion (n = 4)

A patient in the minimal ablative margin < 5 mm group developed a severe infection, which improved with antibiotics. In the group with ablative margins ≥ 5 mm, one patient developed a bronchopleural fistula and was treated with continuous closed thoracic drainage. After 3 weeks, the patient recovered and was discharged.

#### Case 1

**Fig. 5:**
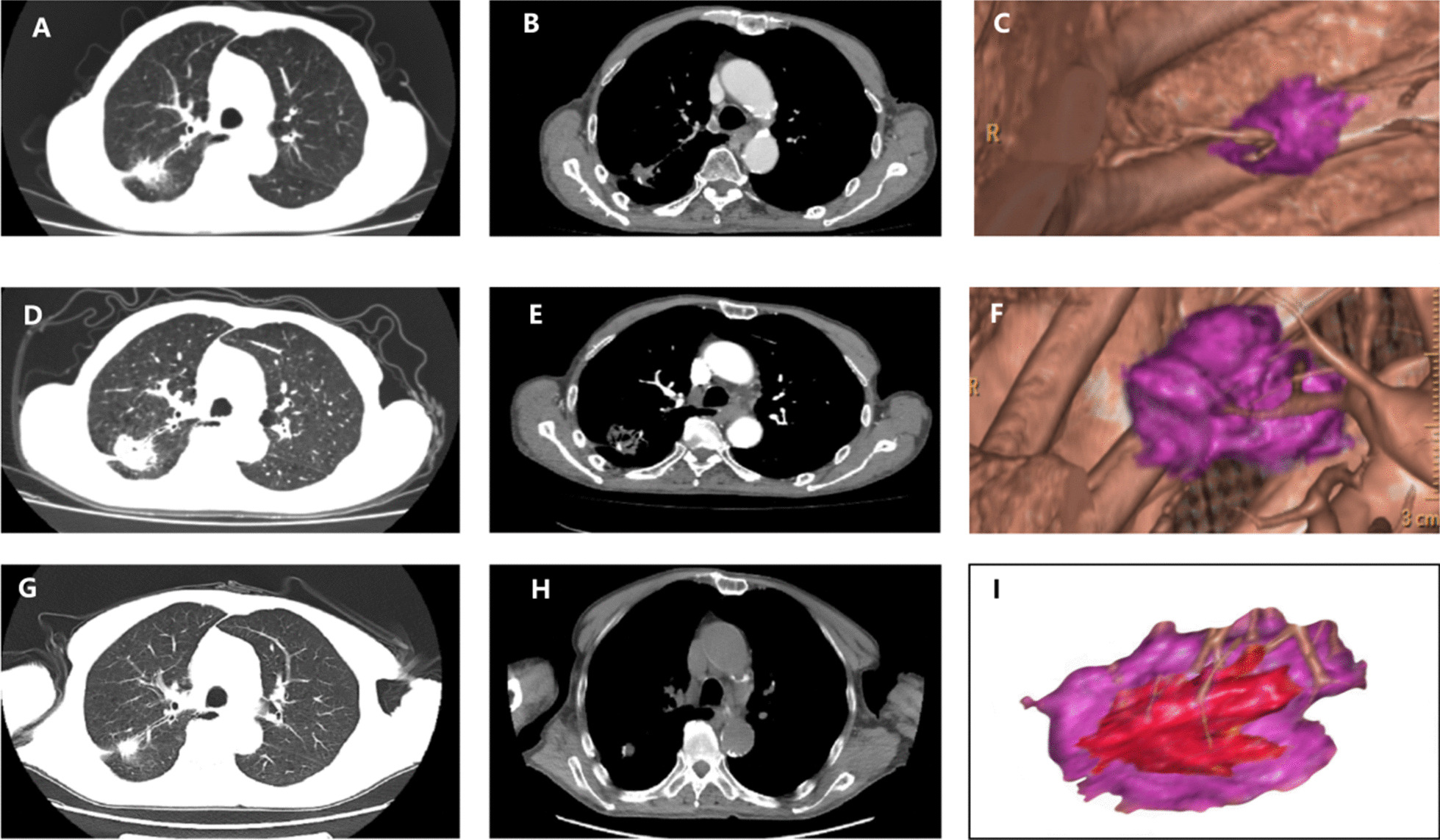
**a**, **b** One lesion was clearly seen in the lung window and mediastinal window, which was pathologically confirmed as squamous cell carcinoma by biopsy. **c** 3D reconstruction revealed the size and margin of the tumor (purple color). **d**, **e** 2 days after ablation, CT showed no pneumothorax and pleural effusion, and the ablation zone was visible. **f** 3D reconstruction revealed the ablation zone and spatial relationships of the tumor (purple color). **g**, **h** 10 months after MWA, the ablative zone shrink and fibrosis had developed. **i** The images of pr-ablation (**c**) and postablation (**f**) were fused and displayed as (**i**). Quantitative measurement shown the minimal ablative margin was > 5 mm

A 73-year-old man was admitted to the hospital for cough lasting half a year. The CT examination revealed a neoplasm in the right lung, and biopsy pathology confirmed squamous cell carcinoma. The patient had smoked for a long time, had poor lung function, and refused surgery and SBRT. Image-guided microwave ablation of the lung cancer was performed with a power of 40 W and an ablation time of 8 min. The CT examination 1 week after ablation revealed no significant pneumothorax or pleural effusion. At the follow-up after discharge, CT showed no recurrence at the 10th month. We reconstructed the pre- and postablation CT to find that the ablation zone completely covered the lesion and the minimal ablative margin was > 5 mm (Fig. 5).

#### Case 2

**Fig. 6:**
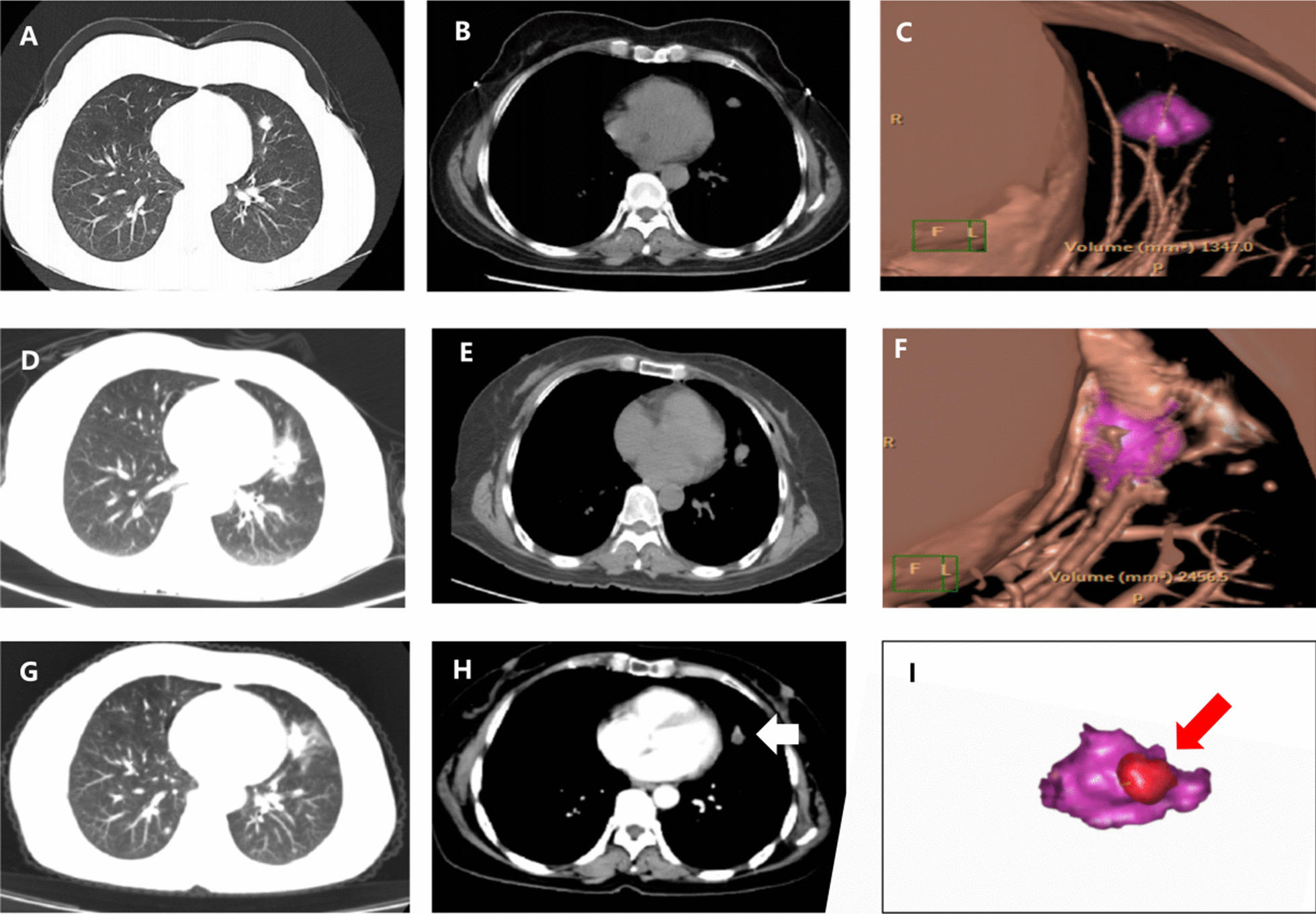
**a**, **b** One lesion was clearly seen in the lung window and mediastinal window, which was pathologically confirmed as adenocarcinoma by biopsy. **c** 3D reconstruction revealed the size and margin of the tumor (purple color). **d**, **e** 3 days later, CT showed no pneumothorax and pleural effusion, and the ablation zone was visible. **f** 3D reconstruction revealed the ablation zone and spatial relationships of the tumor (purple color). **g**, **h** 4 months after MWA, there was enhancement in the ablation zone (the white arrow), which was considered as recurrence. **i** The images of pr-ablation (**c**) and postablation (**f**) were fused and displayed as (**i**). Quantitative measurement shown the minimal ablative margin was < 5 mm (the red arrow)

A 63-year-old woman's health examination revealed a neoplasm in the left lung, which was confirmed by biopsy pathology to be adenocarcinoma. This patient refused the surgery. Microwave ablation was performed with a power of 40 W and a time of 5 min. The patient was followed up at 4 months after the procedure, and enhancement in the ablation zone was found, which was considered recurrence. We reconstructed her pre- and postablation CT and found that the minimal margin of the ablation zone was less than 5 mm (Fig. 6).

## Discussion

In our study, univariate and multivariate survival analyses showed that minimal ablative margin and tumor size were independent prognostic factors for local recurrence and progression. The ROC curve calculated the cut-off value of relapse-related margin to be 4.9 mm. Some previous studies have also considered that 5 mm is a safe margin for lung cancer ablation [18, 25]. Therefore, we divided the patients into two groups based on a margin of 5 mm.

In previous reports on RFA, tumor size, morphology, blood vessels and ablative margin were independent risk factors for local progression [18]. In recent years, microwave ablation has been increasingly used for lung cancer because this method is not affected by arterial or bronchial heat sinks. Wolf et al. [26] and Zhong et al. [27] found that tumors > 3 cm in diameter were associated with recurrence, and Lu et al. [28]believed that a tumor diameter > 4 cm was closely related to treatment failure.

In our study, we found that a tumor diameter greater than 3 cm was strongly associated with recurrence. We also found a negative correlation between tumor size and minimal ablative margin. We considered that malignant tumors had lobulation and/or spicules, so an increased tumor size needs a larger ablation zone. This increased the difficulty of achieving complete ablation.

Compared with a minimal ablative margin of < 5 mm, the safe margin of ≥ 5 mm was associated with a longer local tumor progression interval and disease-free survival. Therefore, it is essential to achieve a sufficient ablative margin. In addition, we observed complications, but the minimal ablative margin ≥ 5 mm group did not have more ablation-related complications.

Traditionally, complete ablation of the tumor was considered if there was no contrast enhancement in the entire ablated zone. Local progression was indicated by either increases in volume with contrast enhancement or by morphologic changes, such as protrusion from the edge of the ablated lesion or an irregular, scattered, nodular on the margin on CT [18].

Conventional two-dimensional images are limited by the ability to exactly assess the ablative margin, especially in cases with a vertical or oblique dimension. It is difficult to accurately observe the minimal ablative margin with 2D CT scanning. In our study, 50% (18/36) of the patients were assessed to have reached the safety ablative margin on CT scan previously, but the minimum ablative margins were found to be < 5 mm in fusion of 3D reconstructions.

During ablation, we regarded the exudate surrounding greater than 5 mm in the axial images as complete ablation. However, it is imprecise to assess ablative margin only from the 2D CT scanning. After three-dimensional reconstruction, we found that the minimal ablative margin was less than 5 mm in 50% (18/36) of cases by stereoscopic comparison. This revealed the limitation of assessing ablation margin on two-dimensional images.

To the best of our knowledge, only a few studies have measured the ablative margin by 3D reconstruction techniques in the treatment of hepatocellular carcinoma (HCC) with RFA [29–31]. They devised a 12 o’clock–6 o’clock coordinate system that consisted of two numerals: the first numeral indicated the direction on the axial plane from 0 o’clock to 11 o’clock, and the second numeral indicated the direction on the vertical plane from 0 o’clock to 6 o’clock. Each direction line could be drawn on the surface of the tumor similar to longitude and latitude lines. Intersecting points made by each type of line created 62 coordinates. However, it took two radiologists to observe and record the 62 coordinates to detect the minimal margin. Each case took 30 min.

The “Lung Nodule Assessment technique” is now widely used to help radiologists improve nodule detection accuracy with efficiency [32]. This software can show the lesion of the lung in 3D and has pseudocolor. We used this method to provide direct visualization of the tumor shape and quantitatively measure the ablative margin. In addition, this technique can automatically calculate the volume of the index tumor and ablative zone. We can rotate the reconstructed 3D images at different angles and visually observe the minimal ablative margin. This approach is feasible and convenient to assess the ablative margin.

There are some limitations to this study. First, this study was limited by its retrospective nature and small sample size. Second, the follow-up time was not long enough, and there were no data on the median local tumor progression interval or median overall survival. Third, the high-density area around the ablation zone might be reactive hyperemia or inflammation but mistaken for the real ablative zone by radiologists subjectivity.

In conclusion, the feasibility of 3D analysis of ablative margins after MWA for NSCLC has been validated. This technique has the potential to improve the complete ablation rate during NSCLC ablation procedures. The 3D reconstruction technique is a method for evaluating the pre- or postablation margin. This approach can significantly reduce recurrence and improve overall survival. However, we still need large-sample prospective studies to verify the precision of this technique.

## Supplementary Information

**Additional file 1**. Editing Certificate by American Journal Experts co, Ltd.

## Data Availability

All data generated or analysed during this study are included in this published article and its supplementary information files.
